# Ulinastatin Reduces Cancer Recurrence after Resection of Hepatic Metastases from Colon Cancer by Inhibiting MMP-9 Activation via the Antifibrinolytic Pathway

**DOI:** 10.1155/2013/437950

**Published:** 2013-04-23

**Authors:** Bo Xu, Kun-ping Li, Fei Shen, Huan-qing Xiao, Wen-song Cai, Jiang-lin Li, Qi-cai Liu, Lin Jia

**Affiliations:** ^1^Department of General Surgery, Guangzhou First People's Hospital, Guangzhou Medical College, 1 Panfu Road, Yuexiu District, Guangzhou 510180, China; ^2^Experimental Medical Research Center, Guangzhou Medical College, 195 Dongfengxi Road, Yuexiu District, Guangzhou 510182, China

## Abstract

High recurrence of colon cancer liver metastasis is observed in patients after hepatic surgery, and the cause is believed to be mostly due to the growth of residual microscopic metastatic lesions within the residual liver. Therefore, triggering the progression of occult metastatic foci may be a novel strategy for improving survival from colon cancer liver metastases. In the present study, we identified an anti-recurrence effect of ulinastatin on colon cancer liver metastasis in mice after hepatectomy. Transwell cell invasion assays demonstrated that ulinastatin significantly inhibited the *in vitro* invasive ability of colon cancer HCT116 cells. Moreover, gelatin zymography and ELISA analysis showed that MMP-9 activity and plasmin activity of colon cancer HCT116 cells were inhibited by ulinastatin, respectively. Furthermore, *in vivo* BALB/C nu/nu mice model indicated that ulinastatin effectively reduced recurrence after resection of hepatic metastases from colon cancer. The optimum timing for ulinastatin administration was one week after hepatectomy. Taken together, our findings point to the potential of ulinastatin as an effective approach in controlling recurrence of hepatic metastases from colon cancer after hepatectomy via its anti-plasmin activity.

## 1. Introduction

 Prognosis of colon cancer is closely associated with the presence of hepatic and other distal metastases. Hepatic metastasis from colon cancer is a typically differentiated adenocarcinoma with limited metastatic foci uncomplicated by other metastases [1]. Therefore, hepatic metastasis from colon cancer is commonly surgically removed, as complete surgical removal is needed for long-term survival or cure [2, 3]. However, hepatectomy can only resect large macroscopic metastatic loci. It is not possible to remove micrometastatic loci that are not detected with current staging methods. It is estimated that a recurrence of 80%–90% is observed within 2 years after hepatectomy, and 40% of these cases are localized to the liver [4, 5]. The cause is believed to be mostly due to the growth of residual microscopic metastatic lesions within the residual liver. Following partial hepatectomy, liver regenerative mechanisms also create a biological environment that accelerates growth of minute residual intrahepatic carcinoma cell foci, such as from colon cancer, that are not detected by hepatectomy [6–9]. Theses foci contribute to local recurrence of cancers. This promotional effect is dependent on paracrine stimulation [10]. Therefore, triggering the progression of occult metastatic foci may be a novel strategy for improving survival from colon cancer liver metastases.

Matrix metalloproteinases (MMPs) are zinc-dependent endopeptidases which are capable of degrading many types of extracellular matrix (ECM) proteins that are involved in the process of tissue remodeling due to various pathologic conditions, including inflammatory diseases, tumor cell invasion, and angiogenesis. Previous studies demonstrated that MMPs, in particular MMP-2 and MMP-9, are released from the liver after hepatectomy [11, 12]. During the late phase of liver regeneration, the effect of MMP activity is correlated to breakdown and remodeling of ECM. The remodeling process may provide scaffolding for tumor cell implantation [13–17]. Consequently, we hypothesized that hepatectomy-induced elevations in MMPs contribute to the growth of metastatic colonic carcinoma in the liver.

Ulinastatin, urinary trypsin inhibitor (UTI) is a multivalent Kunitz-type serine protease inhibitor found in human urine and blood [18]. Various serine proteases such as trypsin, chymotrypsin, neutrophil elastase, and plasmin are reportedly inhibited by UTI. Based on the multivalent nature of protease inhibition, UTI is considered to be an innate anti-inflammatory regulator. Clinically, UTI is used for treatment of pancreatitis, acute circulatory disorder, and regulation of hemodynamic stability during surgical stress. In addition to its anti-inflammatory properties, UTI has been reported to inhibit tumor invasion in an *in vitro* assay and cancer metastasis in an *in vivo* mouse model [19–23]. Serine protease plasmin degrades a variety of ECM components and activates several MMPs, including MMP-9. Therefore, we investigated whether UTI, via its antiplasmin activity, had an antirecurrence effect on liver metastasis in a mouse model of colon cancer following hepatic surgery.

## 2. Materials and Methods

### 2.1. Ethics Statement

The study was carried out in compliance with the guidance suggestion of Animal Care Committee of Guangzhou Medical College (Permit number: SYXK (Guangdong) 2010-0104). The study protocol was approved by the ethics committee of Guangzhou Medical College (Permit number: 2011-54). All surgeries were performed under sodium pentobarbital anesthesia, and all efforts were made to minimize suffering.

### 2.2. Cells and Animals

Colon cancer HCT116 cell lines were obtained from Shanghai Institute of Cell Biology at the Chinese Academy of Sciences (Shanghai, China). Recombinant plasmid pRC/CMV2-luc+ were provided by Professor Qi-Cai Liu (Guangzhou Medical University, Guangzhou, China) [19–23]. Four-to-six week old female nude mice (BALB/C nu/nu) were purchased from the Sun Yat-Sen University Laboratory Animal Center (Guangzhou, China). The mice were maintained in the laboratory for animal experimentation in a specific pathogen-free environment, in laminar air-flow conditions with a 12-hour light-dark cycle, and at a temperature of 22°C to 25°C. All animals had free access to standard laboratory mouse food and water. 

### 2.3. Transwell Invasion Assay

Seven treatment groups for the transwell cell *in vitro* invasion assay were established: (1) low-dose ulinastatin (L-UTI), 800 U/mL ulinastatin (Techpool Bio-Pharma Co., Ltd., Guangzhou, China); (2) high-dose ulinastatin (H-UTI), 8000 U/mL ulinastatin; (3) recombinant IL-1*α* (10 ng/mL) (PeproTech EC Ltd. London, UK) + plasminogen (10 ug/mL) (Athens Research & Technology, Inc. Athens, Georgia, USA); (4) IL-1*α* (10 ng/mL) + plasminogen (10 ug/mL) + L-UTI (800 U/mL); (5) IL-1*α* (10 ng/mL) + plasminogen (10 ug/mL) + H-UTI (8000 U/mL); (6) the broad spectrum MMPs inhibitor GM6001 (0.8 nmol/L) (BioVision Inc., Milpitas, CA, USA); (7) negative controls without reagents.

 The *in vitro* invasion assay was performed using BioCoat Matrigel Invasion Chambers (Becton, Dickinson and Company, Franklin Lakes, NJ, USA) according to the manufacturer's instructions. Briefly, log-phase HCT116 cells were harvested, digested with 0.25% trypsin (Gibco, USA), centrifuged, and adjusted with fresh medium to a cell density of 1 × 10^6^ cells/mL. HCT116 cells (1 × 10^5^ cells) were seeded into Matrigel precoated transwell chambers, consisting of polycarbonate membrane with 8 *μ*m pores. Transwell chambers were then placed in 24-well plates containing basal medium (control), or medium supplemented with L-UTI (800 U/mL), H-UTI (8000 U/mL), 10 ng/mL IL-1*α* + 10 ug/mL plasminogen, 10 ng/mL IL-1*α* + 10 ug/mL plasminogen + L-UTI, 10 ng/mL IL-1*α* + 10 ug/mL plasminogen + H-UTI, or 0.8 nmol/L GM6001, and 500 ul Dulbecco's Modified Eagle's Medium (DMEM, Gibco, USA) containing 10% fetal bovine serum (Gibco, USA) was placed in the lower chamber as a chemoattractant. Following incubation in a saturated humidity incubator containing 5% CO_2_ at 37°C for 24 h, the upper surfaces of transwell chambers were wiped with a cotton swab, and invasive cells were fixed in 4% formaldehyde and stained with 2% ethanol containing 2% crystal violet. The invading cells were counted in five random microscope fields (200x).

### 2.4. Gelatin Zymography Experiment

The effect of ulinastatin on MMP-9 activity in the treatment groups was evaluated using gelatin zymography. Log-phase HCT116 cells (2 mL, 1 × 10^5^ cells/mL) were seeded into 6-well culture plates. When cells grew to 70%–75% fusion, serum-free medium was added to each group for washing three times. Devices were incubated with serum-free medium (control), or serum-free medium supplemented with L-UTI (800 U/mL), H-UTI (8000 U/mL), 10 ng/mL IL-1*α* + 10 ug/mL plasminogen, 10 ng/mL IL-1*α* + 10 ug/mL plasminogen + L-UTI, 10 ng/mL IL-1*α* + 10 ug/mL plasminogen + H-UTI, or 0.8 nmol/L GM6001, for 24 hours in a humidified atmosphere with 95% air and 5% CO_2_ at 37°C. After incubating, the supernatants were collected and were subjected to SDS-PAGE using 10% acrylamide gels containing 0.1% gelatin. After electrophoresis for 2 h at 95 V, the gels were then incubated for 45 min at room temperature in a solution (50 mM Tris-Cl pH 7.6, 10 mM CaCl_2_, 20 mM NaCl, and 1 uM ZnCl_2_) containing 2.5% Triton X-100 twice, before being transferred to activation buffer (50 mM Tris-Cl pH 7.6, 10 mM CaCl_2_, 20 mM NaCl, 1 uM ZnCl_2_) for overnight incubation at 37°C. Gels were then stained with 0.25% Coomassie Brilliant Blue R-250 in 45% methanol and 10% acetic acid and then briefly destained in 10% acetic acid and 30% methanol. The locations of gelatinolytic enzymes were visualized as clear bands on the blue background. 

### 2.5. Enzyme-Linked Immunosorbent Assay (ELISA) Detection of Ulinastatin's Effects on Plasmin Expression

Cells were seeded into 96-well plates at a cell density of 1 × 10^4^ cells/well in a DMEM medium supplemented with 10% FBS which was covered and incubated overnight at 37°C, and the next day, the culture medium was changed to serum-free DMEM. Groups were assigned as described above. After 48 h, the supernatants were collected, and the concentrations of plasmin were measured using ELISA kits (CUSABIO BIOTECH, China) according to the manufacturer's instructions. 

### 2.6. HCT116 Cells Were Stably Transfected with Luciferase

HCT116 colon cancer cells were transfected with recombinant plasmid carrying luciferase gene according to the Lipofectamine 2000^TM^ reagent manual (Invitrogen, Carlsbad, CA, USA). Stable clone cells were used to establish the spleen-injected liver metastatic nude mouse model. Twenty-four h after transfection, 0.1 g/mL to 1.0 g/mL concentration gradients G418 (Invitrogen) were added. Culture medium was changed every 2 days, keeping G418 selection until resistance of single cell clone. Selection of resistant G418 single-cell clones to 96-well plates, and then gradually expanding culture; when subculture to the fifth generations, Luciferase Assay System was used to test the fluorescein enzyme activity of cells. Before testing, cells were seeded into 24-well plates at a cell density of 1 × 10^5^ cells/well. Devices were incubated for 24 hours in a humidified atmosphere with 95% air and 5% CO_2_ at 37°C. After incubation, 1x cell Lysis Buffer (Beyotime, China) were added for cracking cells, and then centrifuged (12000 rpm, 4°C) for 10 min. 10 ul supernatant in each well was added to the 96-well whiteboard plates; 50 ul luciferase substrate was added to each well. After leaving 2 s, continuous reading of 10 s Relative Luciferase Units (RLU), and retaining the high RLU value cell clones passages every time subsequently luciferase activity of cells was detected each ten generations and gradually eliminated lower RLU value cloned cell until passaging to the 40th generation; stable expression of the luciferase clone HCT116-luc+ is positive clones.

### 2.7. Assay for Experimental Liver Metastasis

Mice were fasted 24 hours prior to surgery and deprived of water 12 hours prior to surgery. Fluorescein-labeled HCT116 cells transduced with pRC/CMV2-luc+ were cultured. Log-phase cells were harvested with 0.25% trypsin, centrifuged, and adjusted with fresh medium to a cell density of 1 × 10^6^ cells/mL. Mice were anaesthetized with an intraperitoneal injection of 1% pentobarbital (Gbico, USA) at a dose of 45 mg/kg. Following sterilization, the mice were incised about 10 mm on the left subcostal, the peritoneum was opened, and the spleen was exposed over the peritoneum; the cell suspension of 5 × 10^6^/100 ul of human colon cancer HCT116 cells that transduced with pRC/CMV2-luc+ was injected into the lower pole of the splenic capsule using a 29 G needle. Cotton swabs containing 75% ethanol were firmly placed on the spleen injection site for hemostasis and killing of cancer cells that extravasated the spleen, so as to prevent intraperitoneal seeding and metastasis. The spleen was returned to the abdominal cavity, and the abdomen was closed. 

### 2.8. Bioluminescence Imaging to Monitor Tumor Development

Following injection of fluorescein-labeled HCT116 cells transduced with pRC/CMV2-luc+, tumor growth in the nude mouse liver metastasis model of colon cancer was observed by bioluminescence technology (Night OWL^II^ LB983 NC100, Berthold, Germany). When the liver was visualized with cancer cells, the left liver lobe was resected.

### 2.9. Animal Treatment Groups and Collection of Liver Specimens

After hepatectomy, mice were randomly assigned to early-stage, advanced-stage, and negative control groups. Ulinastatin (50 KU/kg) was administered via the tail vein immediately after hepatectomy for 1 term (1 term = 7 d) in early-stage treatment. Advanced-stage treatment mice were administered with ulinastatin (50 KU/kg) for 1 term via the tail vein one week after hepatectomy. Negative controls were injected with physiological saline via the tail vein immediately after hepatectomy for 1 term. Mice were sacrificed twenty-one days after hepatectomies; liver tissues were collected.

### 2.10. Quantitative Stereological Analysis

Livers in treated and control animals were removed and fixed in 4% formaldehyde and liver tissue was cut into 1.5 mm slices; a mean of 11 ± 2 slices were examined per animal by using quantitative stereological analysis [24]. 

### 2.11. Detection of Cell Proliferation and MMP-9 Level

Tissues obtained 0.5–1 cm^3^ were fixed with 4% formaldehyde, embedded in paraffin using standard procedures, and routinely cut into sections (5 um-thick). All sections were mounted on gelatin-coated glass slides. Histological diagnosis of liver metastasis was ascertained by hematoxylin and eosin staining. Immunohistochemical staining was performed using rabbit antihuman Ki-67 monoclonal antibody (Abcam, Cambridge, MA, USA) and rabbit antihuman MMP-9 monoclonal antibody (BioWorld, Atlanta, GA, USA) according to IHC World (http://www.ihcworld.com/). Cells with brown or brownish yellow granules were considered as positive. Fromowitz's scoring [25] was used to assess the staining of Ki-67 and MMP-9: (i) 0, no coloured stain; 1, light-yellow stain; 2, yellow-brown stain; 3, brown or dark brown stain; (ii) 0, area of positive staining < 5%; 1, the positive stained area constituted 5%–24%; 2, the positive stained area constituted 25%–49%; 3, the positive stained area constituted 50%−74%; 4, area of positive staining > 75%. Sum scores (score (i) + score (ii)) < 3 were considered weakly positive, 3–5 points were considered positive, and >5 was considered strongly positive. Ten fields were randomly selected under an optical microscope at a magnification of 200x, and the mean value of the ten scores was the final point total.

### 2.12. Statistical Analyses

All data were expressed as mean ± standard deviation (SD), and all statistical analyses were performed using SPSS version 18.0 (SPSS Inc.; Chicago, IL, USA). Differences in measurements were compared using one-way analysis of variance (ANOVA), quantitative stereological analysis in treated and control animals were performed by the Mann-Whitney *U* test, and Fisher's least significant difference (LSD) method was employed to compare the means between groups. A *P* value < 0.05 was considered statistically significant. 

## 3. Results 

### 3.1. Anti-Invasive Ability of Ulinastatin on Colon Cancer Cells

**Figure 1:**
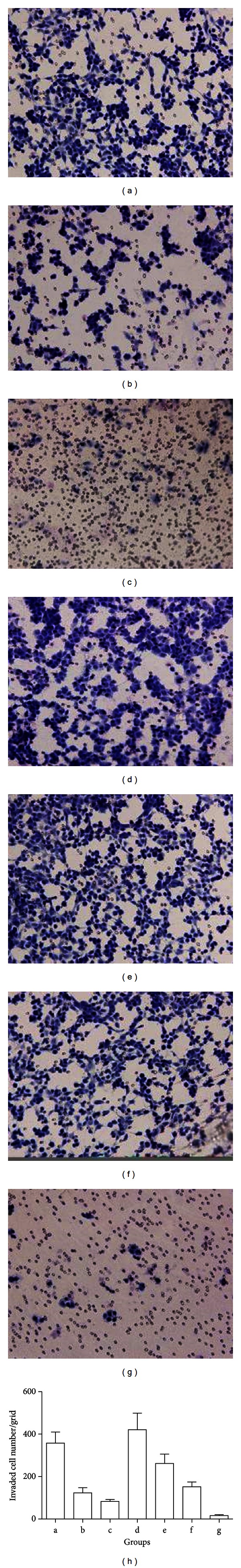
Transwell *in vitro* invasion assay detects the effect of ulinastatin on the invasive ability of colon cancer cells. (a) Control group without reagents; (b) low-dose ulinastatin (L-UTI) group, 800 U/mL ulinastatin; (c) high-dose ulinastatin (H-UTI) group, 8000 U/mL ulinastatin; (d) IL-1*α* (10 ng/mL) + plasminogen group (10 *μ*g/mL); (e) IL-1*α* (10 ng/mL) + plasminogen (10 *μ*g/mL) + L-UTI group (800 U/mL); (f) IL-1*α* (10 ng/mL) + plasminogen (10 *μ*g/mL) + L-UTI group (8000 U/mL); (g) GM6001 group (0.8 nmol/L).

To evaluate the effect of ulinastatin on colon cancer cells, invasion assays were performed. As IL-1*α* has been implicated in promoting plasminogen activity, we chose IL-1*α* + plasminogen groups as positive control. Transwell cell *in vitro* invasion assays demonstrated that L-UTI, H-UTI, and GM6001 significantly inhibited the *in vitro* invasive ability of colon cancer HCT116 cells in artificial basement membranes. Compared with negative controls, the highest inhibition was observed in the GM6001 groups, sequentially followed by the H-UTI and L-UTI treatment groups (*P* < 0.05). The invasive ability of HCT116 cells in the IL-1*α* + plasminogen groups was significantly greater than controls (*P* < 0.05). There were no significant differences in the invasive ability of HCT116 cells among the IL-1*α* + plasminogen, IL-1*α* + plasminogen + L-UTI, and IL-1*α* + plasminogen + H-UTI groups (*P* < 0.05) (Figure 1).

### 3.2. MMP-9 Activity Inhibition by Ulinastatin

**Figure 2:**
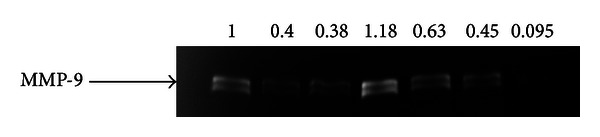
Gelatin zymography detects the effect of ulinastatin on MMP-9 activity. Horizontal ordinate: (1) control group without reagents; (2) low-dose ulinastatin (L-UTI) group, 800 U/mL ulinastatin; (3) high-dose ulinastatin (H-UTI) group, 8000 U/mL ulinastatin; (4) IL-1*α* (10 ng/mL) + plasminogen group (10 *μ*g/mL); (5) IL-1*α* (10 ng/mL) + plasminogen (10 *μ*g/mL) + L-UTI group (800 U/mL); (6) IL-1*α* (10 ng/mL) + plasminogen (10 *μ*g/mL) + L-UTI group (8000 U/mL); (7) GM6001 group (0.8 nmol/L).

Next, we examine the effect of ulinastatin on MMP-9 activity. Gelatin zymography showed that the group with the highest activity of MMP-9 expression was the IL-1*α* + plasminogen group (*P* < 0.05), sequentially followed by activity in the negative controls, IL-1*α* + plasminogen + L-UTI groups, IL-1*α* + plasminogen + H-UTI groups, L-UTI treatment groups, H-UTI, and GM6001 groups. The IL-1*α* + plasminogen + H-UTI groups had lower MMP-9 expression than the IL-1*α* + plasminogen + L-UTI groups (*P* < 0.05) (Figure 2).

### 3.3. Effect of Ulinastatin on Antiplasmin Activity

**Figure 3:**
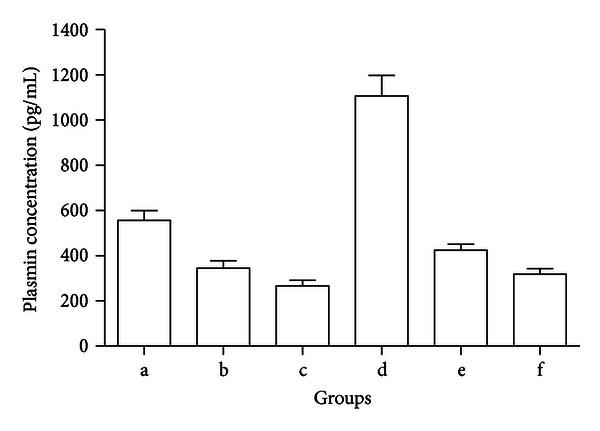
Inhibition of ulinastatin on plasmin expression. Horizontal ordinate: (a) control group without reagents; (b) low-dose ulinastatin (L-UTI) group, 800 U/mL ulinastatin; (c) high-dose ulinastatin (H-UTI) group, 8000 U/mL ulinastatin; (d) IL-1*α* (10 ng/mL) + plasminogen group (10 *μ*g/mL); (e) IL-1*α* (10 ng/mL) + plasminogen (10 *μ*g/mL) + L-UTI group (800 U/mL); (f) IL-1*α* (10 ng/mL) + plasminogen (10 *μ*g/mL) + L-UTI group (8000 U/mL). Longitudinal coordinate: plasmin level (pg/mL).

Next, we asked whether the mechanism of inhibition of MMP-9 activity by ulinastatin was due to interference with plasminogen/plasmin system. The highest plasmin expression was observed in the IL-1*α* + plasminogen groups (*P* < 0.05), sequentially followed by expression in the negative control, IL-1*α* + plasminogen + L-UTI groups, IL-1*α* + plasminogen + H-UTI, L-UTI treatment, and H-UTI treatment groups. (Figure 3).

### 3.4. HCT116 Cells with Stably Expressing Firefly Luciferase

**Figure 4:**
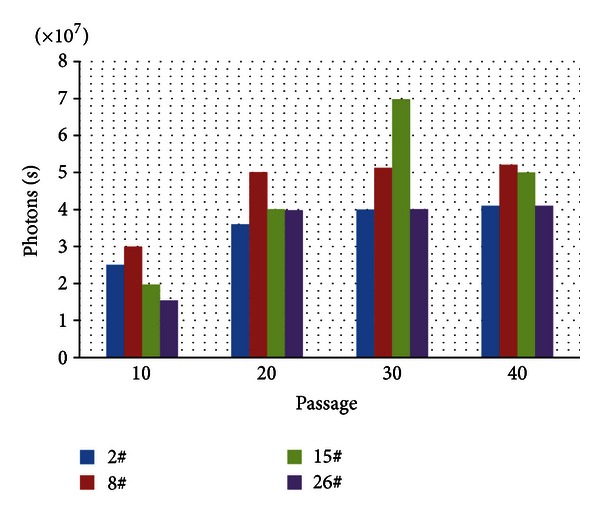
The luciferase activity data in each group.

**Figure 5:**
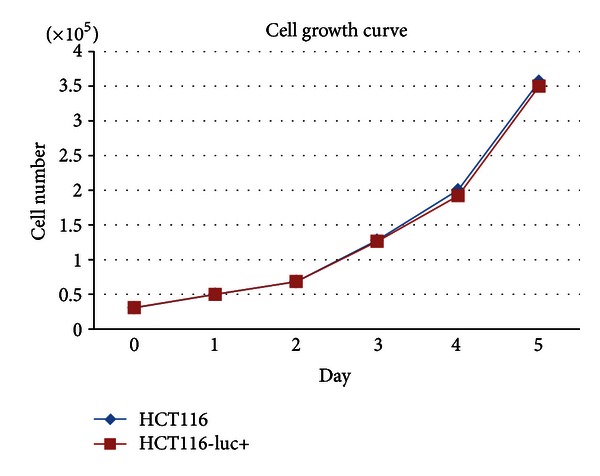
The cell growth curve of HCT116 and HCT116-luc+.

In order to further study the effect of UTI *in vivo*, HCT116 colon cancer cells were transfected with recombinant plasmid carrying luciferase gene according to the manual, and four clones were isolated after antibiotic selection, HCT116-luc+-2, HCT116-luc+-8, HCT116-luc+-15, and HCT116-luc+-26. As expected, 4 clones were maintained for at least 40 passages *in vitro*, the luciferase activity was shown in Figure 4. Clone number 8 (HCT116-luc+-8) was chosen for further characterization because it presented more stable and higher activities. We also observed the growth of HCT116-luc+ and HCT116 cells by cell counting. They had similar growth trend and cell morphology. The stability of cells in the build process does not have a greater impact on the growth characteristics of the HCT116-luc+ (Figure 5).

### 3.5. *In Vivo* Bioluminescence, Animal Treatment, and Collection of Tumor Specimens

**Figure 6:**
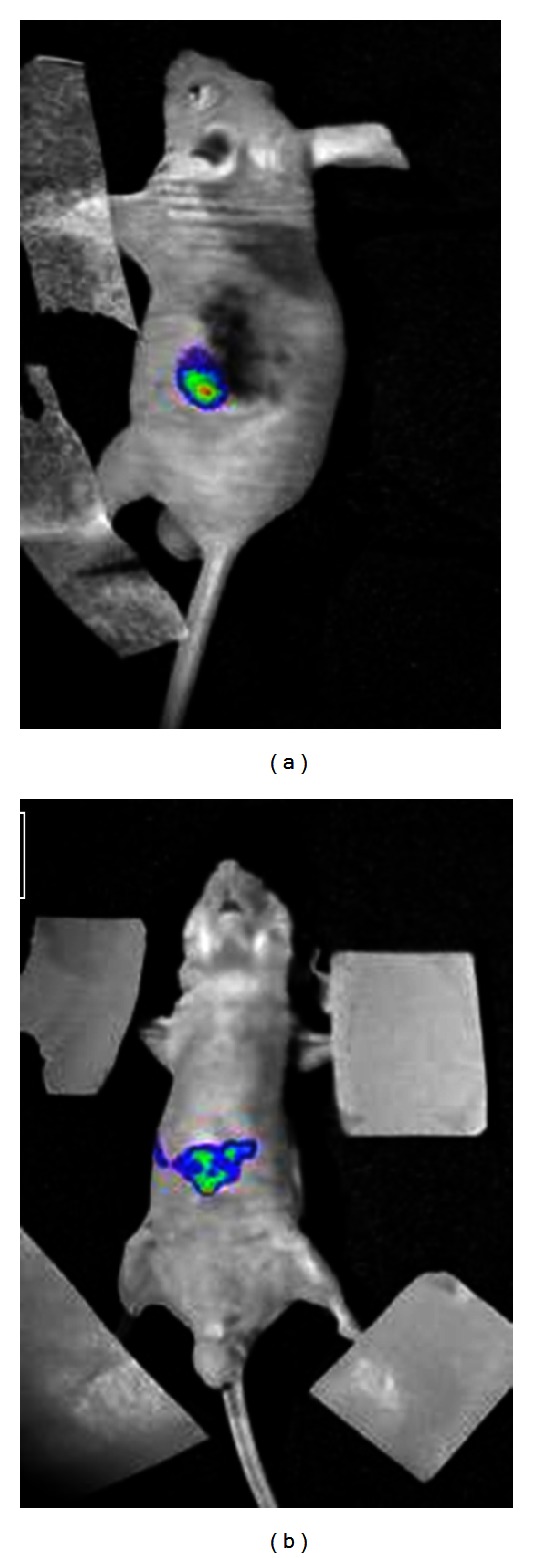
Colon cancer HCT116 cell transduced with pRC/CMV2-luc+ that metastasized in nude mice. (a) Visualization of spleen 2 days after inoculation with tumor cells; (b) visualization of liver 14 days after inoculation with tumor cells.

**Figure 7:**
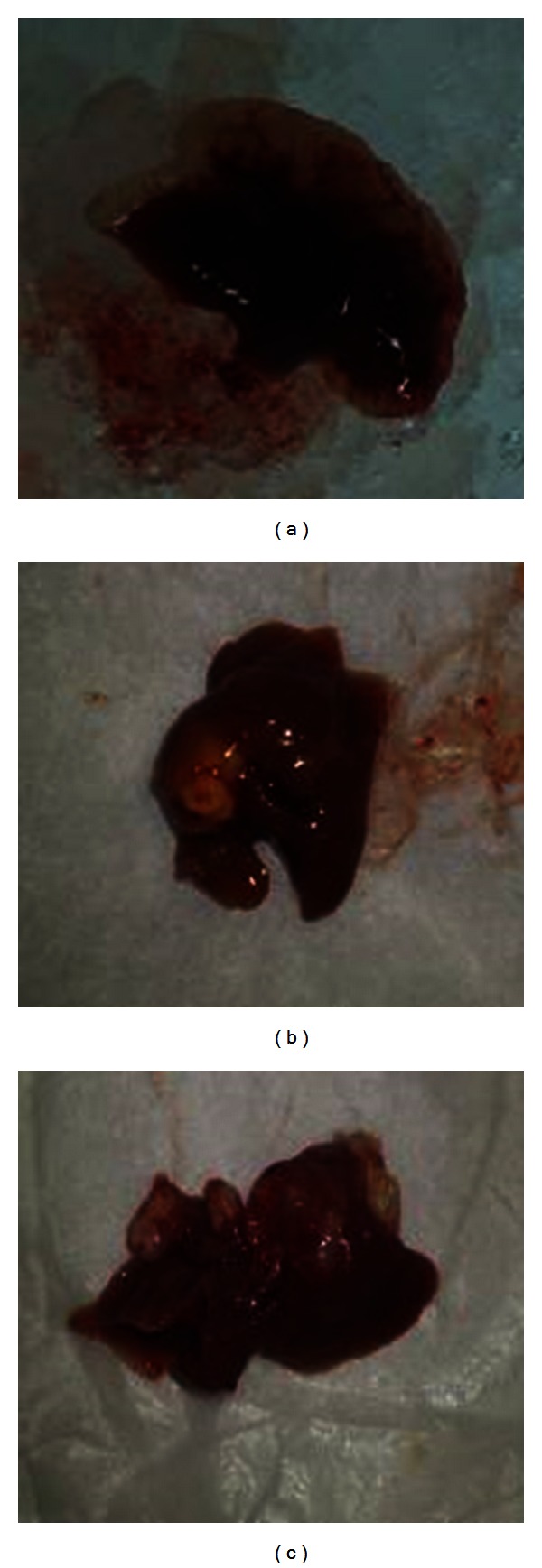
Different types of metastatic tissues in liver from colon cancer. (a) Diffuse liver metastasis; (b) nodular liver metastasis; (c) mixed liver metastasis.

After injection of cancer cells, the mice were imaged by bioluminescence technique. As shown in Figure 6, 14 days after inoculation with tumor cells, the liver was observed with image, and 33 out of 35 nude mice had colon cancer metastases to the liver. After hepatectomy, mice were randomly assigned to early-stage, advanced-stage and negative control groups for treatment. Twenty-one days after hepatectomy, the mice were killed, and liver metastasis was confirmed pathologically. Postsurgical pathological examination revealed nodular hepatocellular carcinoma in 10 cases (30.31%), diffuse hepatocellular carcinoma in 15 cases (45.45%), and mixed hepatocellular carcinoma in 8 cases (24.24%) (Figure 7).

### 3.6. The Influence of Ulinastatin on Hepatic Metastases

**Figure 8:**
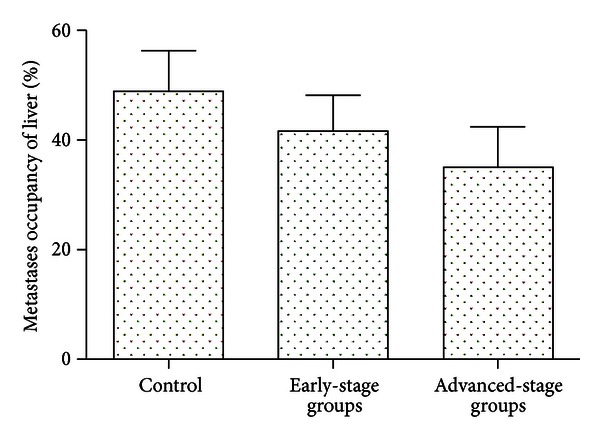
The influence of ulinastatin on hepatic metastases. Percentage of liver occupied by neoplasm 35 days after induction of colorectal liver metastases. Thirty-three mice went through hepatectomy, and mice were randomly assigned to early-stage, advanced-stage, and negative control groups for treatment.

As shown in Figure 8, the percentage of liver metastases ((tumor volume/total liver volume) × 100%) in early-stage treatment (41.64% ± 6.56%) and advanced-stage treatment groups (35.18% ± 7.22%) was significantly different from control groups (48.91% ± 7.40%) (*P* < 0.05), and the lowest average percentage metastases were in the advanced-stage treatment groups (*P* < 0.05). 

### 3.7. Effect of Ulinastatin on the Expression of Ki-67 and MMP-9 in Tumor Specimens

**Figure 9:**
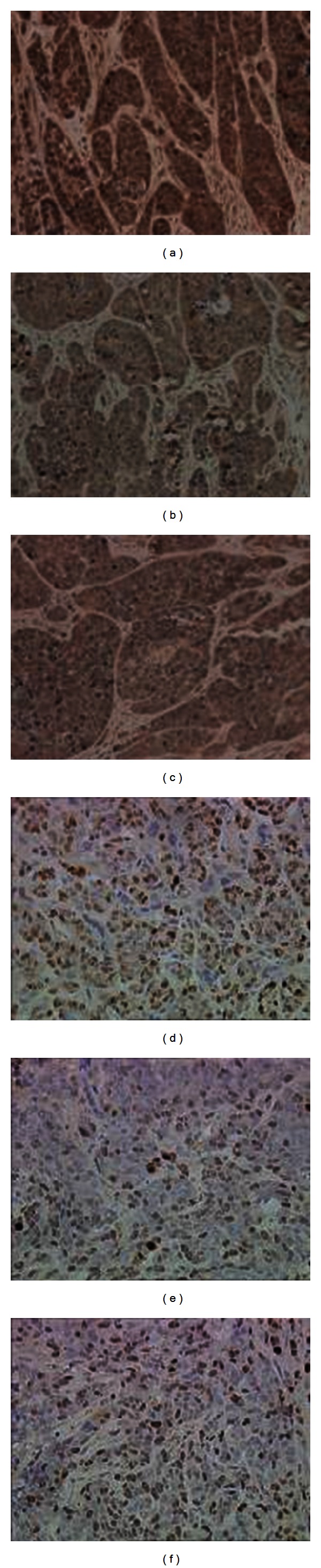
Immunohistochemistry of MMP-9 and Ki-67 in liver tissues. (a) Immunohistochemistry staining of MMP-9 in liver tissues from negative controls; (b) immunohistochemistry staining of MMP-9 in liver tissues from the advanced-stage treatment groups; (c) immunohistochemistry staining of MMP-9 in liver tissues from the early-stage treatment groups; (d) immunohistochemistry staining of Ki-67 in liver tissues from negative controls; (e) immunohistochemistry staining of Ki-67 in liver tissues from the advanced-stage treatment groups; (f) immunohistochemistry staining of Ki-67 in liver tissues from the early-stage treatment groups.

We examined the effects of ulinastatin on liver metastasis in a mouse model of colon cancer. Ki-67 positive expression was significantly different (*P* < 0.05) among early-stage treatment (4.53 ± 0.96), advanced-stage treatment (3.54 ± 1.01), and negative control groups (5.92 ± 0.71) (Figure 9 and Table 1). The results indicated that ulinastatin treatment significantly suppressed growth of colon cancer metastasis in the liver. Next, we examined the effects of ulinastatin on the expression of MMP-9 in tumor specimens. Significant differences in MMP-9 positive expression were detected among the early-stage treatment (5.55 ± 0.86), advanced-stage treatment (4.71 ± 0.99), and negative control groups (6.32 ± 0.72) (*P* < 0.05). Positive expression of Ki-67 and MMP-9 in the early-stage and advanced-stage treatment groups was lower than that in the negative control group, with the lowest expression in the advanced-stage treatment group (Figure 9 and Table 1). Our results showed that growth of liver metastasis from colon cancer was associated with elevated tumor tissues MMP-9, and treatment of ulinastatin significantly reduces MMP-9 expression.

## 4. Discussion

In the past 10 years, the number of market available new chemical entities (NCEs) developed by western countries exhibited a yearly declining trend. This may be explained by one or more of the following three causes: (i) high costs for development of novel drugs currently, development of a novel NCE costs a mean 0.89 billion dollars in America; (ii) US Food and Drug Administration (FDA) strengthened review of new drugs prolongs processing times, development of a novel drug requires at least 15 years from the time of research project approval until market available; (iii) novel drugs that are successfully market-available still encounter unexpected adverse effects. Therefore, “discovering new potential in old drugs” can overcome the problems of long developmental periods, high risks, and high costs to develop novel drugs and provide more treatment approaches for clinical practice. Encouraging examples of new uses for old drugs include thalidomide, originally developed to treat morning sickness, as an effective treatment for multiple myeloma; losartan, a blood pressure controlling medication, used to prevent aortic dissection in patients with Marfan syndrome; sildenafil, originally a cardiovascular disease drug, used for male erectile dysfunction [26]. Therefore, it is relevant for us to explore new indications for conventional agents to prevent tumor recurrence. 

Based on the multiple effects of protease inhibition, the clinical basis for using UTI as described: (1) treatment of acute pancreatitis and shock [27, 28]; (2) *in vivo *protective effect against ischemia-reperfusion injury in the liver [29], kidney [30], heart [31], and lung [32] via the actions of its radical scavenging elements [33]; (3) regulation of hemodynamic stability during surgical stress [34–37]. In addition, UTI may prevent both the growth of cancer via the suppression of cell division as well as the invasion of cancer through the modulation of intracellular signaling and the inhibition of proteases that degrade the ECM [19, 38]. These studies suggest that UTI is a candidate anticancer drug, although further studies are required.

Different types of cytokines/growth factors are released during various stages of liver regeneration. Liver regeneration involves two stages, an early stage that is characterized by an upregulation of numerous factors involved in the restoration of hepatic masses and a late phase involving factors that regulate reestablishment of liver structure. Concentrations of the factors involved in early- and late-phase liver regeneration are modulated by a regeneration signal that dictates liver mass replacement within a given time frame [39]. Many of the factors that are upregulated during liver regeneration are also implicated in tumor growth and recurrence [40–42]. Such factors could include MMPs, specifically MMP-2 and MMP-9 [11]. Most MMPs are synthesized and secreted as inactive zymogens that are activated extracellularly. Pro-MMP-9, an inactive zymogen, is converted to the active form (MMP-9) by a catalytic function [43]. Inuzuka et al. [44] hypothesized that uPA, coexpressed with MMP-9 in colorectal cancers, is responsible for the activation of plasminogen to plasmin. Plasmin activates pro-MMP-3 to MMP-3, which then activates pro-MMP-9 in the promotion of colorectal cancer progression. Since IL-1*α* has been shown to activate pro-MMP-9 by inducing PA-mediated conversion of plasminogen into plasmin [45, 46], we designed a combined treatment of IL-1*α* + plasminogen as positive controls for this study. We examined the effects of ulinastatin on the activation of pro-MMPs mediated by the plasminogen activator/plasmin system in HCT116 cells and a model of colon cancer liver metastases. In this study, we found that treatment with ulinastatin could inhibit the invasive activity and growth of HCT116 colon cancer cells *in vitro* and *in vivo*. To clarify the inhibitory mechanism of tumor cell invasion, we examined the effect of ulinastatin on the activation of MMP-9 and plasminogens. Activation of MMP-9 and plasminogen was inhibited by both ulinastatin and the broad-spectrum MMP inhibitor GM6001. In addition, IL-1*α* and plasminogen accelerated the conversion of pro-MMP-9 into active MMP-9, and ulinastatin blocked the accumulation of active MMP-9. Our data are consistent with the hypothesis that ulinastatin inhibits activation of pro-MMPs in hepatic metastatic colonic carcinoma via the inhibition of plasmin that was converted from plasminogen.

 UTI is considered as an innate anti-inflammatory regulator that is essential for normal liver regeneration and regulation by cytokines and chemokines. Impairment of liver regeneration was observed in UTI^−/−^ mice with high cytokine and chemokine levels [47]. Multiple pharmacological properties that may contribute to inhibition of MMPs during advanced stages of liver regeneration, and there may be a dynamic inhibition-promoting balance that is regulated by inflammatory mediators. Therefore, identification of the optimal timing for drug administration is critical for ensuring ulinastatin inhibition of tumor proliferation and invasion while reducing the adverse effects on liver cell regeneration. DNA synthesis in liver cells does not change 12–14 h after partial hepatectomy in rats. However, 24 h after hepatectomy, rat DNA synthesis reaches a peak followed by a gradual decline. DNA synthesis levels are normal 7–10 days after hepatectomy, much as observed in mice, rabbits, dogs, and humans. Therefore, the best time to use ulinastatin is about one week after hepatectomy. This avoids the time of liver cell regeneration and repair and favors remodeling of the intrahepatic microenvironment. Our results observed that ulinastatin in advanced-stage treatment group had a more efficient inhibition of hepatic metastases compared to the early-stage treatment group. This is likely due to the order that different types of cytokines/growth factors are released during corresponding stages of liver regeneration following hepatectomy. MMPs are released late during liver regeneration and repair, and one week after hepatectomy is when ulinastatin can fully inhibit MMP-9 activation. 

In conclusion, we showed that ulinastatin reduces recurrence after resection of colon cancer hepatic metastases in mice after hepatic surgery by inhibiting MMP-9 activation via the antifibrinolytic pathway. The optimum timing for ulinastatin administration and inhibition of MMP-9 activation is one week after hepatectomy. Further follow-up observations are warranted to identify and improve clinical effective treatments for controlling recurrence of hepatic metastases from colon cancer after hepatectomy.

## Figures and Tables

**Table 1:** Effect of ulinastatin on expression of MMP-9 and Ki-67 in tumor tissues.

Treatment groups	Sample size (*n*)	Protein expression (staining score), mean ± SD	
		MMP-9	Ki-67
Negative control	11	6.32 ± 0.72	5.92 ± 0.71
Early stage	11	5.55 ± 0.86^a^	4.53 ± 0.96^a^
Advanced stage	11	4.71 ± 0.99^a,b^	3.54 ± 1.01^a,b^

^a^ *P < *0.05 versus negative control group; ^b^ *P < *0.05 versus early-stage treatment group.
